# From Reflection to Action: A Reflective Learning Workshop to Bridge Gaps in Pediatric Health

**DOI:** 10.15766/mep_2374-8265.11651

**Published:** 2026-11-10

**Authors:** Amundam Mancho, Brigette Obokhae, Ashley Nmoh, Kendra Moore, Dennis J. Spencer

**Affiliations:** 1 Fellow, Department of Emergency Medicine, Boston Children's Hospital; 2 Graduate Student, Department of Public Health and Preventive Medicine, St. George's University; 3 Medical Student, Duke University School of Medicine; 4 Resident, Department of Pediatrics, The University of North Carolina; 5 Instructor in Pediatrics, Division of Gastroenterology, Hepatology, and Nutrition, Boston Children's Hospital

**Keywords:** Diversity, Equity, and Inclusion, Reflective Learning, Health Equity, Pediatrics, Case-Based Learning

## Abstract

**Introduction:**

Health disparities significantly affect pediatric populations, yet many pediatric trainees and faculty report limited confidence in addressing inequities in clinical and educational settings. Although diversity, equity, and inclusion (DEI) education is increasingly prioritized in graduate medical education, curricula often rely on didactic or case-based formats with limited use of reflective practice. Reflective learning has been shown to foster cultural humility and professional growth but remains underutilized in medical education.

**Methods:**

We developed a 1-hour, interactive, case-based workshop with guided self-reflection. The workshop's goal was to help participants think about their own experiences, learn from others, and discuss DEI in a supportive environment. Grounded in Kolb's experiential learning theory and Mezirow's transformative learning framework, the workshop used pediatric cases, structured reflective prompts informed by Gibbs's reflective cycle, and facilitated small-group dialogue. We delivered the workshop 3 times: once in person at a national pediatric conference and twice virtually. Participants included medical students, residents, fellows, faculty, and program leaders.

**Results:**

Anonymous pre- and postworkshop surveys assessed confidence in recognizing bias, addressing inequities, and applying reflective frameworks. Of 98 participants, 74 had matched responses. Significant improvements occurred across all measured domains (*P* < .05), with moderate to large effect sizes (Cohen's *d* range: −1.03 to −0.68). The largest gains were in participants’ confidence in applying reflective theory and recognizing the role of bias in pediatric health inequities.

**Discussion:**

This scalable, theory-driven workshop integrates reflective practice into pediatric equity education and was associated with meaningful improvements in participants’ self-reported confidence.

## Educational Objectives

By the end of this activity, learners will be able to:

1. Describe key diversity, equity, and inclusion concepts and their relevance to pediatric health care.
2. Use reflective learning to examine how provider bias may contribute to inequities in pediatric health care.
3. Apply practical strategies and insights from reflective learning to recognize and mitigate provider bias and promote equitable pediatric care.

## Introduction

Health disparities continue to affect all populations, and addressing pediatric health disparities is critical given their potential to influence health outcomes across the lifespan. Yet, many pediatric trainees and faculty report discomfort addressing inequities in both clinical and educational settings.^1^ Although diversity, equity, and inclusion (DEI) education is increasingly prioritized in graduate medical education, most curricula rely on didactic or brief case-based formats that lack interactive teaching and implementation of reflective practices.^2,3^ Reflective learning has been shown to promote cultural humility, empathy, and actionable advocacy, yet it remains underutilized in pediatric training.^4^

This educational innovation is designed for pediatric trainees (medical students, residents, and fellows), faculty, and program leaders who are interested in modeling equity-centered care. Prior studies across multiple institutions identified significant gaps in medical trainees’ self-awareness of bias, confidence in discussing microaggressions, and skills for addressing structural inequities.^1,3^ These findings underscore the need for structured opportunities to reflect on personal biases, identify how they affect pediatric care, and develop sustainable strategies for embedding equity in pediatric care and education.

Existing *MedEdPORTAL* resources have made important contributions to advancing pediatric equity education. For instance, Roth et al^5^ developed a social determinant of health (SDOH) curriculum for clerkship students, emphasizing foundational knowledge and patient-level context, Marsh et al^6^ introduced advocacy and SDOH concepts through interactive sessions, and Hoffman et al^7^ presented a project planning tool for community-based advocacy. Our innovation addresses a gap in *MedEdPORTAL* resources by uniquely integrating guided self-reflection with case-based dialogue to promote internalization of DEI principles and their application in clinical and academic contexts. This 1-hour, interactive workshop balances experiential learning with structured discussion, creating a psychologically safe space for participants to engage with DEI concepts such as bias, intersectionality, and cultural humility—elements often missing from prior approaches. Rather than merely absorbing information, reflective learning asks individuals to pause, analyze what happened, consider why it happened, and identify how they can grow or change.^8,9^ This approach translates reflection into action and promotes the application of equity principles. Compared with prior interventions, this approach offers greater depth, learner engagement, and adaptability across institutions. The workshop sought to promote reflective learning that deepens awareness of bias and informs equity-centered action.

## Methods

### Theoretical Frameworks

Our workshop is grounded in Kolb's experiential learning theory and Mezirow's transformative learning framework.^10,11^ It combines real-world pediatric cases with guided reflections to help learners use a structured framework to integrate DEI principles into their reflective practice, apply those reflections to current and future pediatric scenarios, and propose actionable strategies for equity-centered practice. Kolb's model emphasizes learning through active experience, while Mezirow's framework supports critical self-examination that leads to perspective transformation.^10,11^ Both frameworks support processes intended to promote behavioral and attitudinal change in equity education.^10,11^ Our workshop also incorporates collaborative strategy-building and guided reflection prompts grounded in Gibbs's reflective cycle. Gibbs's reflective cycle is a model that guides learners through structured stages of describing, processing, evaluating, analyzing, and concluding what was learned or could have been done differently, followed by action planning for future situations.^12,13^ This approach reinforces the translation of insight into intentional behavioral change and encourages the application of equity principles.

### Design and Development

Our workshop was developed by 2 medical students (BO and AN) and 2 pediatric resident physicians (AM and KM) with longstanding experience in health equity education and advocacy leadership within the American Academy of Pediatrics as well as local, regional, and national leadership roles within the Student National Medical Association. The workshop was reviewed throughout development by 4 clinical content experts representing diverse pediatric specialties and practice settings to support clinical accuracy, relevance, and alignment with current best practices. These faculty members reviewed the cases, learning objectives, discussion prompts, and facilitator materials for educational quality and relevance to pediatric practice. All faculty mentors are recognized experts in health equity with national leadership roles.

We used the Kern 6-step model^14^ to develop, implement, and assess this workshop. In step 1, problem identification and general needs assessment, we conducted a literature review of documented workshops to assess how DEI principles are incorporated into training for pediatricians and pediatric trainees. We also incorporated key content from the 2024 *Pediatrics* report entitled “Diversity, Equity, and Inclusion, Child Health, and the Pediatric Subspecialty Workforce,” which highlighted the need for diverse workforce training in pediatrics.^15,16^ In step 2, we conducted a targeted needs assessment through an informal focus group discussion with approximately 30 Student National Medical Association members to better understand knowledge gaps. In step 3, we drafted the learning objectives with input from pediatric faculty mentors. In step 4, we chose educational strategies, including a slide presentation and an interactive case-based discussion, to promote greater engagement among workshop participants (Appendix A). We implemented this workshop 3 times (step 5). For step 6, we electronically administered evaluation forms via a QR code (ie, pre- and posttests) to assess changes in participants’ confidence and to gather feedback on workshop design and content (Appendices B and C).

### Implementation

Our 1-hour workshop was delivered 3 times: once in person to medical trainees at a national pediatric conference and twice virtually as part of a pediatric trainee workforce development workshop. Audiences ranged from 10 to 100 participants and included medical students, residents, fellows, faculty, and program leadership. Voluntary participation resulted in diverse learner groups from multiple institutions and with varied training backgrounds. We did not control for differences in participant characteristics. Additionally, because participation was anonymous, we could not assess potential repeat participation. Facilitators included AM, BO, AN, and KM.

The introduction, presurvey, and objectives were reviewed in the first 5 minutes of the session. This was followed by a 15-minute overview of key terms, including diversity, equity, inclusion, bias, intersectionality, and cultural humility. No prior knowledge of health equity, health disparities, or related concepts was required. Prior experience with health equity curricula, case-based discussions, or structured reflective exercises was not necessary. Participants’ experiences and knowledge of these terms were then explored in relation to pediatric care. Reflective learning was defined as an individual process in which learners examine their own experiences, thoughts, decisions, and actions to gain a deeper understanding of themselves and improve their future clinical practice.^8,9^

In the first 2 versions of this workshop, participants engaged in two 15-minute, case-based small-group discussions using real-world clinical scenarios addressing bias and disparities in clinical and academic settings. Cases were developed and reviewed by the workshop developers, as described in the Design and Development section, and informed by recurring themes identified during the informal focus group. Each case included discussion questions, reflective prompts, and key take-home messages (Appendix A). Learners examined factors influencing pediatric care, reflected on personal biases, and identified actionable solutions. Groups included 6 to 15 participants, with facilitators promoting inclusive dialogue and psychological safety. For the final virtual workshop, the format was modified to 1 case to allow greater engagement and reflection, with the second case retained as supplemental material. Twenty-five minutes were allotted to case discussion, followed by 15 minutes for the roadmap exercise and 5 minutes for the postsurvey and questions.

Implementation was designed for in-person and virtual delivery. The large in-person session used approximately 1 facilitator per 15 participants, while virtual sessions assigned 1 facilitator per breakout room. Facilitators should have foundational knowledge of DEI concepts, including bias, cultural humility, health disparities, and health equity. In-person requirements included a large room with round tables, audiovisual equipment, reflection materials, and electronic devices for QR code evaluations. Virtual delivery required a platform supporting breakout rooms. Materials included a slide presentation, a reflection worksheet, writing materials, and an optional facilitator guide (Appendices D and E). The facilitator guide provides additional references to support workshop delivery.

### Evaluation and Analysis

We collected evaluation data through anonymous pre- and postworkshop surveys assessing participants’ confidence in their understanding and their perceived ability to address bias and inequities in pediatric health care settings (Appendices B and C). A QR code linked to the pre- and postsurveys was created for each session. For virtual formats, the survey links were posted in the platform chatbox for easy access. Participants created a unique self-identifier that was used to match responses to the postworkshop survey. Survey items were scored on a 5-point Likert scale. Lower scores indicated greater self-reported confidence. Participants also completed open-ended reflection prompts to provide feedback on the workshop's strengths and areas for improvement. Personal electronic devices were used to complete the surveys.

Quantitative data were analyzed using descriptive statistics and paired-sample comparisons to assess changes in mean scores across learning objectives. Open-ended responses were reviewed alongside the quantitative results to identify participant feedback on workshop content and delivery.

## Results

### Participant Demographics

Ninety-eight participants completed the preworkshop survey, and 97 completed the postworkshop survey. Seventy-four participants had matched pre- and postworkshop responses and were included in paired analyses. Participants included medical students (n = 54, 55%), residents (n = 34, 35%), fellows (n = 3, 3%), and attendings, faculty, or emeritus faulty (Table 1).

**Table 1.**
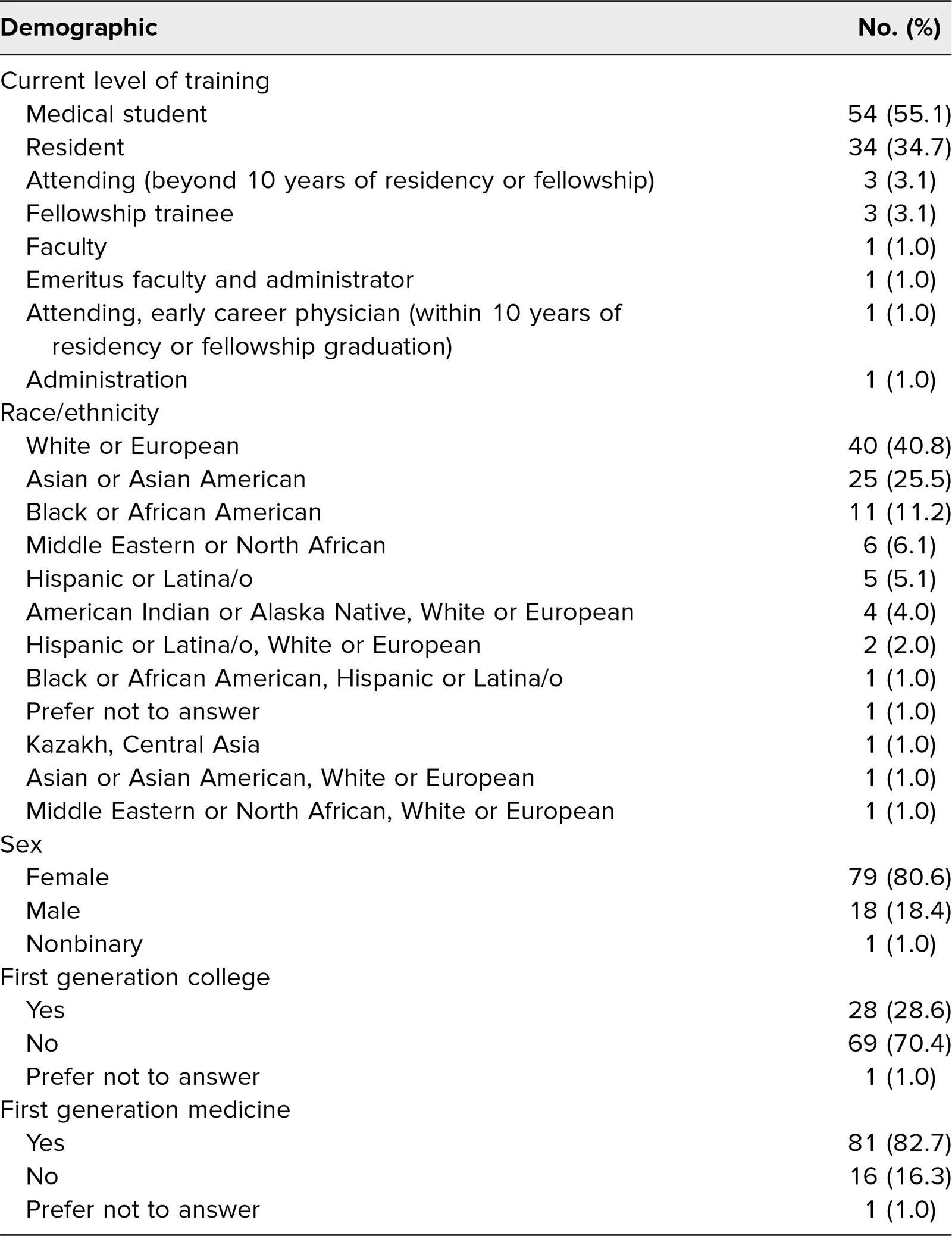
Demographic Data for Workshop Participants

### Quantitative Data

Postsession survey completion rates were 99% (n = 97). Likert responses were coded from 1 (strongly agree) to 5 (strongly disagree), with lower scores indicating stronger agreement. In the full group analysis (n = 97), mean Likert scores decreased across all outcomes, indicating stronger agreement following the workshop, with pre-post differences ranging from −0.96 to −0.59. Effect sizes were moderate to large (Cohen's *d* range: −1.03 to −0.68). The largest gains were in participants’ confidence in their ability to apply reflective theory and recognize the role of bias in pediatric health inequities (Table 2).

Within the paired analysis, 74 participants had matched pre-post identifiers. Item-level paired analyses included participants with complete responses for each item (n = 64-74). Statistically significant improvements were observed in 6 of 6 measured domains (*P* < .05). Across all domains, postworkshop mean Likert scores were lower than baseline, indicating greater agreement after the workshop (Cohen's *d* range: −0.89 to −0.62).

**Table 2.**
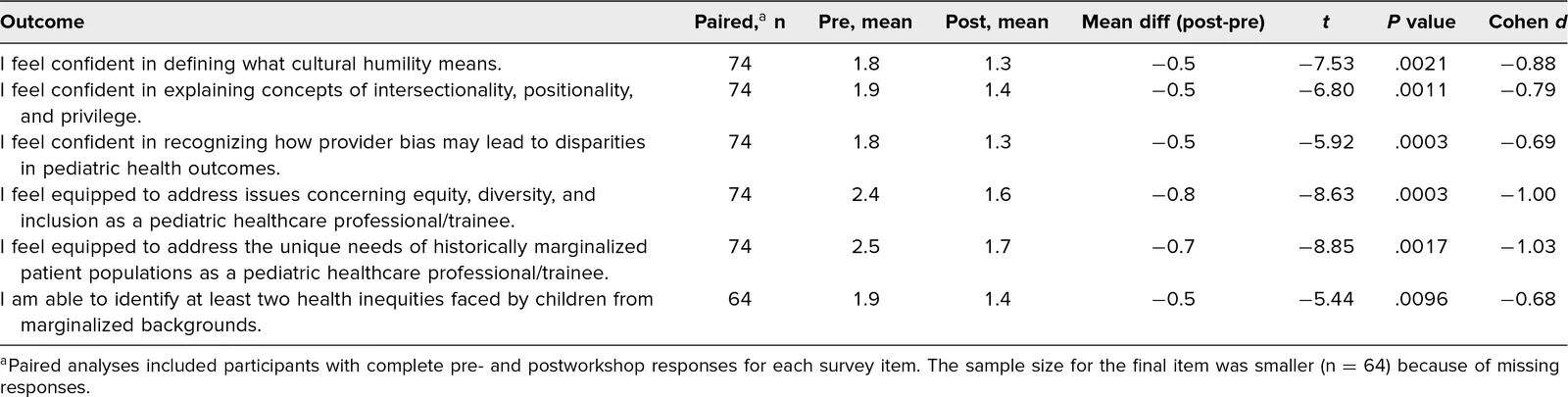
From Reflection to Action: A Reflective Learning Workshop to Bridge Gaps in Pediatric Health—Comparison of Pre- and Postworkshop Self-Reported Learning Outcomes

### Qualitative Data

Ninety-five respondents provided open-ended feedback about the workshop. When asked what they liked, most respondents highlighted the interactive, case-based format and opportunities to learn from one another. One participant reported that the cases “prompted really meaningful discussion in small groups,” while another found the discussions “helpful to learn from peers’ perspectives.” Participants also valued having space to discuss experiences that may otherwise remain unspoken, including “microaggressions in the workforce.” They described the cases as relevant to their experiences, with 1 participant characterizing them as “very familiar and broadly recognizable cases, unfortunately all too common in our systems.” Participants also responded positively to guided reflection and the discussion environment, describing presenters as “very personal and nonjudgmental” and the setting as an “open, supportive environment.”

Participants also shared constructive suggestions when asked how the workshop could be improved. Twenty-five respondents (26%) identified the need for additional time, particularly for discussion, reflection, and deeper exploration of the cases. One participant explained, “I know you were using the time you were given, but I think it's so important to have a little more time to discuss the cases and get into the deeper reflective questions.” Participants also asked for more opportunities to move from reflection toward practice. Their suggestions included “actionable steps on how to advocate for our patients/peers,” “tools on how to address inequities among patients and healthcare peers,” and the “ability to practice forming action plans and opportunities to discuss responses to different situations.”

## Discussion

Grounded in Kolb's experiential learning theory^10^ and Mezirow's transformative learning framework^11^, our workshop conceptualizes reflection not merely as an outcome but as a recurring process for addressing pediatric health inequities. Gibbs's reflective cycle further operationalizes this process by guiding learners through structured stages of describing, processing, evaluating, analyzing, and action planning, reinforcing the translation of insight into action.^12,13^

Unlike most curricula that introduce SDOH or advocacy concepts in isolated or didactic formats, our innovation integrates facilitated reflection using a reflective learning approach. Early implementation data demonstrated significant improvements in participants’ self-reported confidence discussing equity-related concepts and applying reflective approaches to pediatric care, suggesting that this workshop may serve as a scalable foundation for equity-centered pediatric education.

However, these findings should be interpreted as evidence of initiating reflective learning rather than as evidence of sustained perspective transformation or changes in clinical practice. This workshop did not evaluate participants’ subsequent behaviors and interactions in future patient encounters. Although the workshop incorporated elements of transformative learning, it is more appropriately viewed as initiating reflective and transformative processes rather than as producing transformation itself. Mezirow describes transformative learning as an iterative process involving critical reflection, reassessment of previously held assumptions, and the gradual reconstruction of meaningful perspectives over time. A single educational session is unlikely to achieve these longer-term outcomes, particularly among experienced clinicians who may have encountered similar clinical scenarios previously.

In the first 2 offerings of this workshop, participants completed 2 real-world pediatric cases with accompanying self- and group-reflections. Our qualitative findings indicated that participants wanted more time for discussion, reflection, and deeper case exploration within the 60-minute workshop. In response to this feedback, we modified the final workshop to review a single case within the 60-minute session. When time permits, we recommend a 90- to 120-minute format to allow for more robust discussion and reflection for 2 cases. An additional case is provided in Appendix D for facilitators using the extended format.

Additionally, in the first 2 offerings, the reflection questions, survey questions, facilitator guide, and manuscript included topics and principles that were not explicitly introduced in the workshop content. The final version of this module refines its focus on DEI principles, which are clearly defined throughout the workshop and associated materials; provides learners with structured frameworks for reflection and action planning; more intentionally guides both self- and group-reflective practices; and removes superfluous information that distracts from the presentation's objectives. In later versions, the workshop title was also updated to align with the objectives and content; by including “Reflection to Action,” the workshop intentionally incorporated more time for learners to translate reflections into actionable items and offered an explicit framework to support this process. These changes help the workshop more clearly demonstrate how to apply reflective learning to pediatric care while giving learners more time to practice and apply the reflective skills taught.

This innovative module provides a scalable, evidence-informed framework that can be adapted across residency programs and medical education to cultivate reflective practice, enhance learners’ confidence in addressing bias, and strengthen institutional capacity to promote equity and inclusion in clinical training environments. Strengths of this workshop include adaptability, minimal material requirements, and high engagement across in-person and virtual formats.

### Limitations

Limitations include reliance on self-reported outcomes and the absence of long-term behavioral or institutional measures. Our survey assessed objectives shared across learner groups rather than differences by training level. Medical students, residents, and fellows comprised 93% of participants, while faculty and program leaders comprised 7%; thus, pooled results primarily reflect trainee outcomes. The small faculty sample precluded meaningful subgroup analysis.

Participants completed baseline assessments using their existing understanding of bias and reflective practice. As the workshop expanded this understanding, participants may have applied a higher standard when rating themselves afterward, potentially narrowing pre- and postworkshop differences and underestimating the observed change. Among faculty, learning may also reflect reassessment of prior assumptions rather than increased confidence, which our design cannot distinguish.

Finally, participation was voluntary, potentially introducing selection bias among individuals with a greater interest in health equity. Future implementation within a single institution or required curriculum could assess adaptability across settings and populations.

### Future Directions

Future development should prioritize assessment design to better account for learners’ differing experience levels. Medical students and residents develop foundational skills in recognizing and responding to bias, while fellows bring greater clinical and teaching experience, and faculty and program leaders may participate as prospective facilitators. Our shared survey assessed objectives common across groups but did not distinguish developmental stages or differences in the depth and complexity of knowledge application. Future assessments could stratify participants by training level, including fellows as a distinct group, and incorporate more specific, scenario-based items of increasing complexity to assess application in ambiguous situations. Comparing performance with self-reported confidence could determine whether perceived confidence aligns with demonstrated understanding. A retrospective pre/post format could also reduce differences in participants’ frames of reference.

We acknowledge that the current survey is limited in its ability to detect advanced changes in knowledge application and transformation. Future versions could incorporate developmentally aligned assessments, measures of bias recognition and application, and longitudinal follow-up to evaluate behavioral change. Three-month assessments could examine the use of reflective practices, barriers to application, and changes in clinical practice and learning environments. Larger samples and qualitative assessments could further explore the depth of reflection and transformative learning. Overall, this workshop provides a feasible, theory-driven pilot model for equity-centered reflection and a foundation for further development of equity-centered education in pediatrics.

## Appendices


Reflective Learning Workshop.pptxPresurvey.docxPostsurvey.docxFacilitator Guide.docxReflection Document for Participants.docx
*All appendices are peer reviewed as integral parts of the Original Publication.*

## References

[1] Tervalon M, Murray-García J. Cultural humility versus cultural competence: a critical distinction in defining physician training outcomes in multicultural education. J Health Care Poor Underserved. 1998;9(2):117-125. 10.1353/hpu.2010.0233 10073197

[2] Chung AS, Cardell A, Desai S, et al. Educational outcomes of diversity curricula in graduate medical education. J Grad Med Educ. 2023;15(2):152-170. 10.4300/JGME-D-22-00497.1 PMC10150806 37139216

[3] Weissman JS, Betancourt J, Campbell EG, et al. Resident physicians’ preparedness to provide cross-cultural care. JAMA. 2005;294(9):1058-1067. 10.1001/jama.294.9.1058 16145026

[4] Kumagai AK, Lypson ML. Beyond cultural competence: critical consciousness, social justice, and multicultural education. Acad Med. 2009;84(6):782-787. 10.1097/ACM.0b013e3181a42398 19474560

[5] Roth C, Prudhomme A, Griese K, Beyl R, Patrick-Esteve J. Social determinants of health curriculum for the pediatric clerkship. MedEdPORTAL. 2024;20:11458. 10.15766/mep_2374-8265.11458 PMC11518917 39473434

[6] Marsh MC, Supples S, McLaurin-Jiang S, Brown CL, Linton JM. Introducing the concepts of advocacy and social determinants of health within the pediatric clerkship. MedEdPORTAL. 2019;15:10798. 10.15766/mep_2374-8265.10798 PMC6376941 30800998

[7] Hoffman BD, Rose J, Best D, et al. The Community Pediatrics Training Initiative project planning tool: a practical approach to community-based advocacy. MedEdPORTAL. 2017;13:10630. 10.15766/mep_2374-8265.10630 PMC6338167 30800831

[8] Mann KV. Reflection's role in learning: increasing engagement and deepening participation. Perspect Med Educ. 2016;5(5):259-261. 10.1007/S40037-016-0296-Y PMC5035282 27638389

[9] Colomer J, Serra T, Cañabate D, Bubnys R. Reflective learning in higher education: active methodologies for transformative practices. Sustainability. 2020;12(9):3827. 10.3390/su12093827

[10] Kolb DA. Experiential Learning: Experience as the Source of Learning and Development. Prentice Hall; 1984.

[11] Mezirow J, Taylor EW, eds. Transformative Learning in Practice: Insights from Community, Workplace, and Higher Education. Jossey-Bass; 2009.

[12] The Open University. Gibbs' reflective cycle. OpenLearn: learning to teach: becoming a reflective practitioner. Accessed September 26, 2026. https://www.open.edu/openlearn/education-development/learning-teach-becoming-reflective-practitioner/content-section-6.2

[13] The University of Edinburgh. Reflection Toolkit: Gibbs' reflective cycle. Published October 15, 2024. Accessed January 8, 2026. https://reflection.ed.ac.uk/reflectors-toolkit/reflecting-on-experience/gibbs-reflective-cycle

[14] Thomas PA, Kern DE, Hughes MT, Chen BY, eds. Curriculum Development for Medical Education: A Six-Step Approach. 3rd ed. Johns Hopkins University Press; 2015.

[15] Orr CJ, Leslie LK, Schaechter J, et al. Diversity, equity, and inclusion, child health, and the pediatric subspecialty workforce. Pediatrics. 2024;153(suppl 2):e2023063678S. 10.1542/peds.2023-063678S PMC10852199 38300010

[16] Leslie LK, Orr CJ, Turner AL, et al. Child health and the US pediatric subspecialty workforce: planning for the future. Pediatrics. 2024;153(suppl 2):e2023063678B. 10.1542/peds.2023-063678B 38299999

