# Supplementary material for: From Reflection to Action: A Reflective Learning Workshop to Bridge Gaps in Pediatric Health
Source: MedEdPORTAL. 2026 Nov 10;22:11651. doi: 10.15766/mep_2374-8265.11651 (PMC13749098; doi:10.15766/mep_2374-8265.11651)
Supplement: Supplementary file 1 [file mep_2374-8265.11651-s001.zip › B. Presurvey.docx]

**Appendix B: Pre-Survey and Evaluation Form**

Pre-Workshop survey to be administered to participants prior to the start of the workshop.

**Personal Identifier**

To protect your privacy, you will create a unique identifier utilizing the combination provided below:

1. Last 2 letters of your last name

2. Last 2 numbers of your cell phone

Please use the same identifier in the pre and post survey

____________________________

**Demographic Information**

1. **What is your current level of training?**
   - Pre-Medical Student
   - Medical Student
   - Doctoral Candidate (PhD Candidate)
   - Resident
   - Fellowship Trainee
   - Attending, early career physician (within 10 years of residency or fellowship graduation)
   - Attending, (beyond 10 years of residency or fellowship)
   - Other Health Professional (RN, NP, PA, PT, OT etc)_______
   - Prefer to Self-Describe_________________
2. **My race or ethnicity is best described as (Please select all that apply or feel free to self-describe how you identify. We are using racial categories based on the US Census and acknowledge that these categories might not be fully representative)**
   - American Indian or Alaska Native
   - Asian or Asian American
   - Black or African American
   - Hispanic or Latina/o
   - Middle Eastern or North African
   - Native Hawaiian or Pacific Islander
   - White or European
   - Prefer to Self-Describe_________________
   - Prefer not to answer
3. **What is your gender?**
   - Female
   - Male
   - Transgender
   - Gender nonconforming
   - Prefer not to answer
   - Other
4. **Do you identify as a member of the LGBTQIA+ Community?**
   - Yes
   - No
   - Prefer not to answer

**5. Do you identify as a first generation college student? A “first-generation college graduate” is defined as a student whose parent/guardian has not received a four-year U.S. bachelor's degree**

- Yes
- No
- Prefer not to answer

**6. Do you identify as a first generation in medicine? This is defined as a person who is attending or attended a medical school and whose parent/guardian did not attend medical school.**

- Yes
- No
- Prefer not to answer

**Survey Questions**

|  | **Strongly Agree** | **Somewhat Agree** | **Neither Agree or Disagree** | **Somewhat Disagree** | **Strongly Disagree** |
| --- | --- | --- | --- | --- | --- |
|  |  |  |  |  |  |
| **I feel confident in my understanding of what diversity, equity and inclusion means.** |  |  |  |  |  |
| **I feel confident in my understanding of the concepts of bias, intersectionality, and cultural humility.** |  |  |  |  |  |
| **I feel confident in my understanding of how provider bias may lead to disparities in child health outcomes.** |  |  |  |  |  |
| **I feel equipped to address issues concerning equity, diversity, and inclusion as a pediatric healthcare professional/trainee.** |  |  |  |  |  |
| **I feel equipped to address the unique needs of historically marginalized patient populations as a pediatric healthcare professional/trainee.** |  |  |  |  |  |
