# Supplementary material for: From Reflection to Action: A Reflective Learning Workshop to Bridge Gaps in Pediatric Health
Source: MedEdPORTAL. 2026 Nov 10;22:11651. doi: 10.15766/mep_2374-8265.11651 (PMC13749098; doi:10.15766/mep_2374-8265.11651)
Supplement: Supplementary file 1 [file mep_2374-8265.11651-s001.zip › C. Postsurvey.docx]

**Appendix C: Post-Survey and Evaluation Form**

Post-Workshop survey to be administered to participants at the end of the workshop.

**Personal Identifier**

To protect your privacy, you will create a unique identifier utilizing the combination provided below:

1. Last 2 letters of your last name

2. Last 2 numbers of your cell phone

Please use the same identifier in the pre and post survey

____________________________

**Survey Questions**

|  | **Strongly Agree** | **Somewhat Agree** | **Neither Agree nor Disagree** | **Somewhat Disagree** | **Strongly Disagree** |
| --- | --- | --- | --- | --- | --- |
|  |  |  |  |  |  |
| **I feel confident in my understanding of what diversity, equity and inclusion means.** |  |  |  |  |  |
| **I feel confident in my understanding of the concepts of intersectionality, positionality, and privilege.** |  |  |  |  |  |
| **I feel confident in my understanding of how provider bias may lead to disparities in neonatal, infant and child health outcomes.** |  |  |  |  |  |
| **I feel equipped to address issues concerning equity, diversity, and inclusion as a pediatric healthcare professional/trainee.** |  |  |  |  |  |
| **I feel equipped to address the unique needs of historically marginalized patient populations as a pediatric healthcare professional/trainee.** |  |  |  |  |  |

**Workshop Evaluation**

1. **How would you rate this workshop overall? (1=poor, 2 = fair, 3=good, 4=very good, 5=excellent)**

**1 2 3 4 5**

2. **How would you rate the case scenarios and its discussions? (1=poor, 2 = fair, 3=good, 4=very good, 5=excellent)**

**1 2 3 4 5**

3. **How likely are you to apply the information you learned in this workshop to your everyday practice? (1=Not at all likely, 2= Somewhat not likely 3= No change 4= Somewhat likely 5 = Extremely likely)**

**1 2 3 4 5**

4. **What did you like about this workshop?**
5. **What suggestions do you have to improve this workshop?**
