# Supplementary material for: From Reflection to Action: A Reflective Learning Workshop to Bridge Gaps in Pediatric Health
Source: MedEdPORTAL. 2026 Nov 10;22:11651. doi: 10.15766/mep_2374-8265.11651 (PMC13749098; doi:10.15766/mep_2374-8265.11651)
Supplement: Supplementary file 1 [file mep_2374-8265.11651-s001.zip › D. Facilitator Guide.docx]

**Appendix D
Facilitator Instructional Guide**

This document contains detailed information about each topic covered in the PowerPoint Slide Deck presentation with instructions and talking points to help facilitators present to the audience and guide discussions.

**Overall Goal**

The goal of this workshop is to create a one-hour reflective learning workshop that reviews key Diversity, Equity, and Inclusion (DEI) principles and allows participants to examine their own perspectives, recognize how bias shapes the training environment and the families they serve, and empowers them to practice inclusive, intentional behaviors that strengthen belonging and help improve pediatric learning spaces and health outcomes.

**Objectives**

1. Describe key diversity, equity, and inclusion concepts and their relevance to pediatric health care.

2. Use reflective learning to examine how provider bias may contribute to inequities in pediatric health	care.

3. Apply practical strategies and insights from reflective learning to recognize and mitigate provider bias	and promote equitable pediatric care.

**Suggested Agenda and Timeline (60 minutes)**

- Introduction, Pre-workshop survey, and Objectives (5 minutes)
- PowerPoint Presentation and Slides
  - Background (15 minutes)
  - Case One (25 minutes)
  - Creating an Action Plan(10 minutes)
- Post-workshop Survey, Questions, and Wrap Up (5 minutes)
- Appendix: Case 2 (Optional if time permits)

**Handouts and Materials**

- Computer setup with audio capability and connection to projector
- Internet
- At least one workshop facilitator depending on audience size. We suggest one facilitator per 15 participants.
- PowerPoint Presentation slide deck
- Pre- and Post- Survey Electronic Evaluation Forms
- Case Studies
- Reflection Document for Participants
- Facilitator Guide

If In-Person presentation,

- A space with full audiovisual capabilities with multiple mics available for facilitators and participants if needed
- 1 printed reflection document handout for each participant (can also consider a QR code within the presentation for users that prefer an electronic version)
- Multiple round tables set up in a crescent round configuration that fosters small group discussions
- Paper and pens

If Virtual,

- The capability to form interactive virtual break-out rooms via online platform
- 1 PDF copy of the reflection document for each participant which can be provided via a link that is posted in the virtual platform chat, emailed to participants prior to the session (can also consider a QR code within the presentation for users that prefer an electronic version)

**Slide Instructions**

**Slide 1:** Title: From Reflection to Action: A Reflective Learning Workshop to Bridge Gaps in Pediatric Health

*The facilitators should introduce themselves to the audience.*

**Slide 2:** Agenda

*The facilitator will present the agenda for the educational intervention. Welcome participants to the session, and set ground rules for safety throughout today’s workshop they are participating in.*

**Slide 3:** Pre-Workshop Survey

*The facilitator will instruct participants to complete the pre-test. Please include QR code for the evaluation form. Please allow at least 2-3 minutes for participants to the scan code on the PowerPoint slide.*

**Slide 4:** Objectives

*The facilitator will present the objectives for the educational intervention.*

**Slide 5:** Background (transition slide)

**Slide 6:** Audience Engagement Checkpoint

*The facilitator will ask the audience “When you hear the terms Diversity, Equity, and Inclusion in the context of Pediatric Health Care, what comes to mind?” Give a 20 second pause, ask the audience for responses for approximately 1 minute.

Facilitator, pause for one minute and allow the audience to raise their hands in the room (or the virtual platform space). You can allow for one to three participants (depending on how you are doing on time and how long the responses are). The facilitator can briefly summarize and hit a key point following each audience member’s response based on what they shared.*

**Slide 7:** Diversity, Equity, and Inclusion

*The facilitator will give a brief introduction to the topics of diversity, equity, and inclusion and their importance in pediatrics. Facilitator can read the definition of diversity from the slide and/or incorporate the definition of the terms as below:*

- ***Diversity:*** *Diversity involves understanding that each individual is unique and recognizing our individual differences. These differences can be along the dimensions of race, ethnicity, ancestry, national origin, immigration status, religion, gender, marital or family status, sexual orientation, gender identity or expression, age, veteran status, disability, or other characteristics.^1^*
- *In the field of pediatrics, diversity can encompass the vast diversity of the patients we take care of and serve as well as their families and other pediatric health professionals and providers that we interact with.*

*The facilitator can read the definition of equity from the slide and/or incorporate the definition of the term as below:*

- ***Health Equity:*** *The state in which everyone has a fair and just opportunity to attain their highest level of health. Achieving health equity requires valuing everyone equally with focused and ongoing societal efforts to address avoidable inequalities, historical and contemporary injustices, and the elimination of health and healthcare disparities.^2,3^*
- *Equity goes the next step further in comparison to equality. It does not give everyone the exact same support regardless of need like equality does. It tends to patients’ and families’ specific strengths and needs to provide them optimal support. It is not a one-size fit all scenario for each person.*

*The facilitator can read the definition of inclusion from the slide and/or incorporate the definition of the term as below:*

- ***Inclusion:*** *Inclusion represents the engagement and participation of diverse individuals in an organization. Initiatives around inclusion recognize the inherent worth and dignity of all individuals, and work to include all into the organization's overall capacity and decision-making.^2,4^*
- *Inclusion is related to yet still separate from diversity. Although an institution or a particular healthcare system can have diversity amongst its patients and staff, that does not mean that it inherently has inclusiveness. Another word for inclusivity is belonging. Do individuals feel like they belong in the grand scope of the community, place, practice, system, etc.*

*After reading through the definitions, we encourage the facilitator to share the information below (or they can share additional updated data that correlates to this topic) to provide audience members with clear examples of DEI initiatives:*

*In early 2025, DEI initiatives and programs in America began to become dismantled in higher education, corporate America, and larger institutions of power created to diversify the national workforce.^5^*

- *Based on the medical literature,* ***racial and ethnic minority children comprise approximately 47-50% of the US pediatric population****, representing about 34.8 million children.^5,6^ This reflects substantial and growing diversity in America's child population.*
- *The demographic composition shows that as of 2019,* ***50% of children younger than 18 years were Hispanic, Black, Asian, or Indigenous****.*
- *The diversity extends beyond race and ethnicity to include multiple dimensions such as country of origin, socioeconomic status, sex and gender identity, disability status, and language.^6^ This increasing diversity has significant implications for pediatric healthcare delivery, as children in the United States are growing up in the most ethnically and racially diverse environment in the nation's history.*

**Slide 8:** Comparing Implicit and Explicit Biases

*The facilitator will give a brief definition about implicit and explicit biases and their importance in pediatrics by pointing out the following:

This slide highlights the difference between implicit and explicit bias in pediatrics. Bias can be described into two broader, larger categories - implicit and explicit biases.*

***Explicit Bias:*** *A belief is explicit if consciously endorsed. Explicit forms of bias include preferences, beliefs, and attitudes of which people are generally consciously aware, personally endorse, and can identify and communicate.^4^*

*Many of you may have heard or even recognize your explicit biases as you are often more consciously aware of them. A more subtle and discrete form of bias is implicit bias.^4,7^*

***Implicit Bias:*** *Implicit bias is the attitude or internalized stereotypes that unconsciously affect our perceptions, actions, and decisions. These unconscious biases often affect behavior that leads to unequal treatment of people based on race, ethnicity, gender identity, sexual orientation, age, disability, health status, and other characteristics.^4,7^*

*In medicine, unconscious bias-based discriminatory practices negatively impact patient care and medical training* *programs, hinder effective communication, limit workforce diversity, lead to inequitable distribution of research funding, impede career advancement, and result in carriers and disparities in the access to and delivery of healthcare services.^11,12^*

*In pediatrics, it can show up subtly—through assumptions about pain, adherence, or parenting capacity.*

*Importantly, implicit bias is a major driver of disparities even when clinicians believe they are providing equitable care.*

*That’s why awareness alone isn’t enough—we need reflection, training, and systems that protect against bias at the point of care.*

**Slide 9:** Personal Identity, Intersectionality, and Cultural Humility

*The facilitator will give a brief definition about topics of personal identity, intersectionality, microaggressions, and cultural humility and their importance in pediatrics by pointing out the following:*

***Personal Identity:*** *is defined as “an individual’s understanding of who they are, shaped by their lived experiences, values, beliefs, roles, and social identities.” ^13^*

*It includes:*

- ***Core values and beliefs*** *(what matters to you, how you see the world)*
- ***Life experiences*** *(culture, family, education, trauma, achievements)*
- ***Social identities*** *(race, ethnicity, gender, sexual orientation, ability, religion, socioeconomic background)*
- ***Roles and relationships*** *(child, parent, clinician, advocate, community member)*
- ***Sense of continuity and agency*** *(how you see yourself over time and your ability to make choices)*

*In pediatrics, personal identity matters because it influences how clinicians interpret patient behavior, how families engage with care, and how children develop their own sense of self within medical, family, and social systems.^13,14^*

***Intersectionality:*** *A term coined by attorney and civil rights activist, Kimberle Crenshaw, that serves as a framework for understanding how various aspects of individual identity such as race, gender, social class, and sexuality, interact to create unique experiences of privilege or oppression.^8, 9^*

***Microaggressions:*** *everyday slights, insults, invalidations, and offensive behaviors experienced by individuals from marginalized groups. These actions often occur during interactions with generally well-intentioned individuals, who may be unaware that their behavior is demeaning or harmful.^10^*

***Cultural Humility*:** Cultural humility involves entering a relationship with another person with the intention of honoring their beliefs, customs, and values. It entails an ongoing process of self-exploration and self-critique combined with a willingness to learn from others.^1^

**Slide 10:** Audience Engagement Checkpoint

*The facilitator will ask the audience, “How has your personal identity influenced your interactions with patients and families?”and should allow approximately 1 minute for audience responses.

Allow the audience to raise their hands in the room (or the virtual platform space). You can allow for one to three participants (depending on how you are doing on time and how long the responses are). The facilitator can briefly summarize and hit a key point following each audience member’s response based on what they shared.*

**Slide 11:** Importance of Understanding Health Inequities and Bias in Medical Training

*The facilitator will discuss the importance of understanding health inequities and bias in medical training by pointing out the following:

Thank you all for sharing the ways in which you have been introduced to DEI teaching throughout your medical education. Read from slide,

“Understanding the impact of bias on health inequities enables providers to build trust, identify barriers to health, and address root causes that promote optimal child health outcomes.”^14^

This is a great segue into our next topic of further expanding upon why it is important to have an understanding of what health inequities and bias are throughout your medical training.*

*I acknowledge that understanding must first come with recognition. Developing comfort with identifying and addressing health inequities and bias cannot be achieved through a single lecture or one-time session. This is a longitudinal process that develops over time through reflection, education, and lived experience. This is quite valuable to our patients and families we serve. The literature shows that diverse, well-supported healthcare teams provide higher quality care and have better patient outcomes. ^15,16^*

**Slide 12:** Audience Engagement Checkpoint

*The facilitator will ask the audience, “How has your medical school, residency program, or institution implemented teaching DEI principles to promote better pediatric health care?”and should allow approximately 1 minute for audience responses.

Allow the audience to raise their hands in the room (or the virtual platform space). You can allow for one to three participants (depending on how you are doing on time and how long the responses are). The facilitator can briefly summarize and hit a key point following each audience member’s response based on what they shared.*

**Slide 13:** Limitations to Traditional DEI Instruction

*The facilitator will discuss limitations of traditional DEI instruction by pointing out the following:

Traditionally, DEI instruction has often been limited to lectures, case-based learning, and standardized patient encounters. These methods do provide some levels of learning but are more superficial in the participant’s level of self-involvement. They often lack components that promote deep self-reflection and implicit bias recognition. In order for us to collectively embody DEI principles, we have to individually look within and recognize the ways that we perpetuate bias. Deep self-reflection allows us to more intimately acknowledge the ways that we operate and navigate with people as well as how those ways could show up to affect patient care. ^4, 17^*

**Slide 14:** What is Reflective Learning?

*The facilitator will emphasize the importance of reflective learning and relevance of the reflective workshop by pointing out the following:

We explicitly defined reflective learning as the practice of processing past or present experience, learning from the outcomes of those experiences, and planning how to better approach similar situations in the future.*

*Psychologist and philosopher John Dewey describes reflection as an “active, persistent, and careful consideration”^17, 18^*

*Rather than only absorbing information in this workshop, reflective learning asks individuals to pause, analyze what happened, consider why it happened, and identify how they can grow or change ^18^*

**Slide 15:** The Power of Reflective Learning

*The facilitator will continue to emphasize the power of reflective learning not just as an educational “add on”, but a skill that directly impacts clinical care, bias awareness, and patient outcomes.*

*The facilitator should walk through the slide starting from left to right to explain how reflective learning works as a process.*

1. *Reflective learning encourages self-examination and critical thinking ^17,18^*
   a. *Self-examination allows you to pause and look inward. This combined with critical thinking helps us to examine our assumptions, reactions, and decision-making.*
   b. *The facilitator can then read from the slide and state how this ultimately helps learners explore their own beliefs and assumptions.*
2. *Reflective learning promotes cultural humility*
   a. *When we reflect, we become more open to learning from our patients and their families. Cultural humility is not about just mastering facts about different cultures, but encourages us to stay curious, recognize our limits, and be willing to adjust how we care for our patients based on their individual needs.*
   b. *The facilitator can then read from the slide and state how this leads to increased awareness of unconscious biases and underlying thought patterns.*
3. *Reflective learning improves learning and reduces bias^18^*
   a. *Over time, reflection helps us to recognize conscious and unconscious patterns that may influence how we treat our patients. This awareness helps us avoid stereotyping and making assumptions and allows us to build better connections with families, show up more intentionally, and make more careful, patient centered clinical decisions.*

**Slide 16:** The Gibbs Reflective Cycle

*The facilitator should first describe The Gibbs Reflective Cycle and the six steps below:

The Gibbs Reflective Cycle is a structured model of reflection developed by Graham Gibbs (1988) to help individuals systematically think about experiences, learn from them, and improve future practice. It is widely used in education, healthcare, and professional development. ^19^*

*The cycle consists of six stages:*

1. *Description – What happened?
    A factual account of the experience without analysis or judgment.*
2. *Feelings – What were you thinking and feeling?
    Reflection on emotions and thoughts before, during, and after the event.*
3. *Evaluation – What was good and bad about the experience?
    Assessment of what worked well and what did not.*
4. *Analysis – What sense can you make of the situation?
    Deeper examination of why things happened as they did, often using theory or evidence.*
5. *Conclusion – What else could you have done?
    Summary of key learning points and alternative actions.*
6. *Action Plan – If it happened again, what would you do?
    Specific steps for improving or changing future practice.*

*The model is cyclical, emphasizing continuous learning by applying insights from reflection to future experiences.*

**Slide 17:** Cases and Reflective Learning (transition slide)

*Suggested dialogue can be the following:*

*We will explore case studies through the Gibbs Reflective Cycle to encourage reflection on experiences, enhance insight, and support meaningful learning for future situations*.

**Slide 18:** Case and Reflective Learning

*The facilitator will present the agenda for the case section of the workshop instructing the audience that they will spend 25 minutes going through an example case. A facilitator will state that a facilitator or audience member will read the case and will encourage the audience to make note of things that stand out to them as the cases are being read. There are a series of self-reflection questions that audience members will have time to reflect on individually and then individual audience members can share their reflections aloud with the audience. Subsequently, there are group reflection questions that small groups can discuss collectively and then will have time to share aloud with the audience what was discussed. Small groups can include 2-6 people depending on the audience size and number of facilitators. After the self and group reflection question responses have been shared aloud, the groups can then discuss aloud with the audience any major key takeaways from the case.*

**Slide 19:** Reflection Cases and Questions

*The facilitator will create a QR code that provides the audience with the link to the reflection document. You can choose to edit the reflection document prior to the presentation to fit your presentation needs or goals. You can share with the audience that the reflection document will have the Gibbs Reflection Cycle, example case and associated questions as well as an additional case (Case 2) that can be reviewed later by audience members after the session for additional practice.*

**Slide 20:** Case #1

*At this time, a facilitator can set a timer for 25 minutes to ensure the presentation moves forward in a timely manner. Either a facilitator or audience member should read the following case study.*

*While the case is being read, audience members should be encouraged to take note of anything that stands out to them from the case.*

“A 5-year-old boy is brought to the ED by his father for lethargy and vomiting. The father has limited English proficiency and smells faintly of marijuana. The resident documents the odor, assumes accidental ingestion, and orders a urine toxicology screen before fully examining the child. The family is placed in an observation room. The interpreter phone is broken. No in-person interpreter is available, and no effort is made to find one.

Hours later, the child becomes hypotensive. Another provider discovers asymmetric pupils and neck stiffness—findings missed on the initial encounter. The family, though distraught, remains calm and asks why no one listened earlier. In sign-out, the resident says, “That family’s always defensive. They’re covering up something,” and warns others to expect a “defensive” family. A team member even suggests flagging them in the EMR for future safety concerns.”

**Slide 21:** Case #1 Self Reflections

*The facilitator will briefly review the questions aloud. They can also emphasize that the word in bold before the start of each question correlates with the part of the Gibbs Reflective Cycle that the question relates to or addresses. The facilitator will allow audience members the time to individually self-reflect. Following the self-reflection, audience members can discuss aloud their responses to the reflection questions. Potential discussion points/answers are displayed below. Facilitators should guide audience members to encourage inclusive dialogue and psychological safety.*

*Remember to keep the conversation centered on the discussion points to provide ample time for the case studies. If the learners overlook any points the facilitator may guide the conversation or state the points.*

| **Self-Reflection Questions** | **Question Guidance** |
| --- | --- |
| How would you describe what just occurred?  Facilitators should note that this question aligns with the **Gibbs Reflective Cycle – “Description” stage**, which focuses on an objective, factual account of what occurred during a situation. | - Facilitators can encourage participants to reflect on the whole picture and describe what happened. - It can be noted that there was not just one mistake, but a sequence of decisions and missed opportunities. - Participants should be encouraged to identify the patterns or assumptions that shaped how this encounter went |
| What explicit or implicit biases did you feel coming up for you?  Facilitators should note that this question aligns with the **Gibbs Reflective Cycle – “Feelings” stage**, which focuses on identifying initial emotional responses before moving into evaluation or analysis. | - Facilitators can invite participants to engage in self-reflection, emphasizing that bias is a common and often unconscious aspect of human cognition and may not present as an overt or emotionally charged response. Bias can surface subtly, influencing what information is prioritized, minimized, or overlooked during clinical decision-making. - Participants should be encouraged to identify potential assumptions related to parenting practices, substance use, language proficiency, credibility of the history provided, and patient or family behavior, and to consider how these assumptions may shape clinical assessment, communication, and care delivery. |
| How would you have displayed cultural humility in this scenario?  Facilitators should note that this question aligns with the **Gibbs Reflective Cycle – “Evaluation” stage**, which emphasizes recognizing what went well and what did not, without assigning blame. This stage helps participants identify gaps and shortcomings in care delivery and systems.  Facilitators should note that this question aligns with the **Gibbs Reflective Cycle – “Analysis” stage**, which encourages participants to make sense of the situation and explore why the events happened as they did. | - As facilitators guide the discussion, participants can be encouraged to place themselves in the moment of care delivery. Practicing cultural humility may involve intentionally slowing down, maintaining curiosity, and acknowledging the limits of one’s own understanding of the patient’s and family’s lived experience. - Participants could also be encouraged to reflect on personal identity and its influence on interactions with patients and families through this scenario. - During the discussion, listen for examples such as active and attentive listening, advocating for appropriate interpreter services, validating the family’s initial concerns, and approaching the family as collaborative partners in the care plan rather than passive recipients of information. |
| Following the completion of this self-reflection, how will you change how you practice in the future?  Facilitators should note that this question aligns with the **Gibbs Reflective Cycle – “Action Plan” stage**, which focuses on considering how future practice may change as a result of reflection. | - Encourage participants to think about one thing they might do differently in the future |

**Slide 22:** Case #1 Group Reflections

*The facilitator will briefly review the questions aloud. They can also emphasize that the word in bold before the start of each question correlates with the part of the Gibbs Reflective Cycle that the question relates to or addresses. The facilitator will allow audience members to form small groups (groups of 2-6 people depending on the audience size) and have time to reflect on the questions as a group. Following the group reflection, audience members from the different groups can discuss aloud their responses to the reflection questions. Potential discussion points/answers are displayed below. Facilitators should guide audience members to encourage inclusive dialogue and psychological safety.*

*Remember to keep the conversation centered on the discussion points to provide ample time for the case studies. If the learners overlook any points the facilitator may guide the conversation or state the points.*

| **Group Reflection Questions** | **Question Guidance** |
| --- | --- |
| What thoughts and feelings came up for you while reading the case?  Facilitators should note that this question aligns with the **Gibbs Reflective Cycle – “Feelings” stage**, which focuses on identifying initial emotional responses before moving into evaluation or analysis. | - The facilitator can encourage participants to pause and reflect on their immediate emotional reactions before moving into analysis or problem-solving. Emphasize that emotions are a meaningful part of clinical reflection and often provide insight into underlying values, assumptions, or discomfort. - Encourage participants to notice a range of responses, including discomfort, frustration, concern, sadness, defensiveness, or even emotional distance. It can be noted that there is no expectation to resolve these feelings, only to acknowledge them as part of the reflective process. |
| What shortcomings did you notice in this case?  Facilitators should note that this question aligns with the **Gibbs Reflective Cycle – “Evaluation” stage**, which emphasizes recognizing what went well and what did not, without assigning blame. This stage helps participants identify gaps and shortcomings in care delivery and systems. | - The facilitator can guide participants to identify areas where care fell short, emphasizing that this was not a single mistake but a series of gaps and missed opportunities. - Encourage participants to name both clinical and non-clinical shortcomings, including missed assessments, early assumptions, failures in communication, language access barriers, and the use of labeling language. - Following the discussion, you can point out that what may seem like a small, missed opportunity can compound and lead to negative health outcomes |
| What sense can you make of this situation? Why do you think this happened? Have you seen similar situations occur in your own clinical settings?  Facilitators should note that these questions align with the **Gibbs Reflective Cycle – “Analysis” stage**, which encourages participants to make sense of the situation and explore why the events happened as they did. | - Facilitators can encourage participants to step back and consider the broader context of this situation. This includes reflecting on how bias, time pressure, cognitive load, hierarchy, and institutional norms may influence clinical decision-making. - Participants should be encouraged to connect this case to their own experiences and recognize patterns instead of viewing this as an isolated event. - Facilitators can normalize that many participants may have witnessed or been part of similar situations, and that naming these patterns is an important step toward change. |
| What else could have been done? Who had the opportunity to intervene? When similar situations have occurred in your own clinical settings, did you intervene? Why or why not?  Facilitators should note that these questions align with the **Gibbs Reflective Cycle – “Conclusion” stages**, which focuses on identifying opportunities for intervention and considering how future practice may change as a result of reflection. | - Facilitators can guide participants to reflect on missed opportunities for intervention at multiple levels of care, including individual clinicians, team members, and system processes. Emphasize that intervention does not have to be dramatic or confrontational. - Participants should be encouraged to reflect honestly on barriers to speaking up, such as hierarchy, fear of conflict, uncertainty, or time constraints. Facilitators can invite participants to consider small, practical actions that could be taken in future situations, including slowing down assumptions, advocating for interpreter services, or challenging labeling language. |

**Slide 23:** Case #1 Key Takeaways

*The facilitator will share the following key takeaways:
- Provider bias can influence assumptions, delay care, and worsen outcomes.
- Health disparities are amplified by structural barriers.
- Negative labeling damages trust and worsens future care.*

*The facilitator can also tie in key points and takeaways that were mentioned by audience members and tie them into these key takeaways as they address each individual take away. The facilitator could even mirror language that was used or described by audience members to better personalize and connect the takeaways to the already discussed points from the audience.*

**Slide 24:** Reflection to Action Quote

*The facilitator can read the quote on the slide “Reflection without action is ultimately as unproductive as action without reflection.” by Robert Kegan. The facilitator can highlight how we have focused quite a bit of our time during today’s presentation on reflective learning, but now we will take the next step beyond reflection and utilize what we have reflected on today to move us to action.*

**Slide 25:** From Reflection to Action

*The facilitator will share with the audience that regarding our current case, we focused primarily on reflection. But the final stage of the Gibbs Reflective Cycle highlights action. We can reflect as much as we would like but will still see no changes as there has been no action following the reflection.*

**Slide 26:** Creating an Action Plan to Address Health Inequities (transition slide)

*The facilitator can share that with that being said, we will now work towards putting our reflections in motion via action. We will now shift the focus of this presentation in this final section towards creating an action plan to address health inequities.*

**Slide 27:** Collaborating to Identify Strategies That Advance Diversity, Equity, & Inclusion in Pediatric Care

*The facilitator should introduce and describe the 5 steps to creating a roadmap to address pediatric health inequities which utilizes the Kern’s six step curriculum development model as its framework. The facilitator can provide a real-life example to the audience that simultaneously shares the origin of this workshop:*

*Kern's six steps framework for curriculum development guided the creation of this workshop*

1) ***Problem Identification and General Needs Assessment:*** *Problem Identification and General Needs Assessment, there is limitation of reflective learning in Pediatric DEI. Pediatric health disparities persist across racial, ethnic, and socioeconomic lines, affecting neonatal, infant, and child outcomes.*
2) ***Targeted Needs Assessment:*** *Using learner centered goals to tailor their current understanding and professional roles to help identify what they need, their comfort with DEI concepts, and recognizing/understanding their bias.*
3) ***Goals and Objectives:*** *Clear workshop objectives that drive the content and assessment. Learners develop specific, measurable objectives during the roadmap.*
4) ***Educational Strategies:*** *Use active, inclusive teaching methods that align with adult learning theory:*

- *Case-based learning: Clinical vignettes illustrating real-world disparities*
- *Reflective learning: Journaling, small-group discussions on DEI-related topics*
- *Role play or simulation: Exploring difficult conversations or navigating biased systems*
- *Action planning: Helping learners identify how to implement equity in their own settings*

5) ***Implementation:*** *Identify facilitators with DEI and pediatric expertise, allocate time for case analysis and reflection, prepare materials (cases, reflection prompts, slide decks, pre/post assessments)*
6) ***Evaluation and Feedback:*** *Implement Pre and Post Surveys to assess knowledge attained throughout the workshop*

*After providing this example, the facilitator can remind the audience to think back on their self and group reflections, key points, and takeaways that have been discussed throughout this presentation. In small groups, they can think through ideas on how to move those reflections into actionable ideas that can be implemented at their programs or institutions. This is a great time to discuss challenges with implementation at certain sites that may no longer be able to openly have DEI initiatives (e.g. labeling it as health innovation and advancement)*

1) *What problems are you hoping to identify to implement change?*
2) *What are the goals and objectives of this change you want to implement on a systems level?*
3) *How do you envision implementing this strategy at your institution or clinical practice? What key stakeholders are needed to implement this change? What educational strategies will support the work towards your goal?*
4) *Once the change is implemented, how will you evaluate or obtain feedback?*

*If time permits, the facilitator can ask the audience members to share what was discussed. This same exercise can be applied to changes or recommendations for personal changes participants may want to implement.*

**Slide 28:** Objectives Revisited

*The facilitator will summarize and review the objectives for the educational intervention that were achieved during this educational workshop.*

**Slide 29:** Questions

*The facilitators should pause to answer any questions from the audience.*

**Slide 30:** Post-Workshop Survey

*The facilitator will instruct participants to complete the post-test. Please include QR code for the evaluation form.*

**Slide 31:** References

**Slide 33:** Appendix: Additional Case (transition slide)

*The facilitator has been provided with an additional case that may be used if the facilitator has time to incorporate it, however it is unlikely that time will permit you to be able to review this case during the presentation. The case has also been provided to the audience members via the reflection document, and the audience can be instructed to complete it on their own following the presentation.*

*There will be a similar structure for the presentation of case 2 as there was for case 1 if the facilitator so happens to utilize case 2 during the presentation. An alternative use for case 2 is if the facilitator gives this presentation on multiple occasions, they can alternate using case 1 or 2 in the main presentation - while still providing both cases on the reflection document for use by the audience members. For example, If the presentation is given in 45-60 minutes, then the presenter can remove the slides 30-34 from the presentation. If the presentation is given with case 2 being the primary case, then slides 18-21 will need to be removed.*

**Slide 34:** Case #2 Description

*Either a facilitator or audience member should read the following case study. While the case is being read, audience members should be encouraged to take notes of anything that stands out to them from the case.*

“Dr. Amina Brooks, a female Afro-Latina pediatric resident, has grown increasingly quiet during team rounds. She used to be excited to work at a hospital that serves a majority Black and Latinx population but now finds herself constantly navigating small microaggressions. She wears her natural hair in styles such as an afro, braids, or twists. She once overheard a fellow whisper, “She’s always doing something different with her hair.” During presentations, she is interrupted more often than her peers and her teaching points are often dismissed as “too detailed”. This rarely happens with other trainees. Amina has started writing notes to reassure herself that she’s not imagining things.

When she shares her discomfort with a chief resident, her concerns are brushed aside that “perception is everything” and that she should “read the room.” She’s later described in a feedback session as “quiet, but intense” and “not very approachable.” Amina begins to question whether she belongs and whether she can thrive in an institution where no one above her looks like her or affirms her presence.”

**Slide 35:** Case #2 Self Reflections

*The facilitator will lead the discussion based on the listed questions. They can also emphasize that the word in bold before the start of each question correlates with the part of the Gibbs Reflective Cycle that the question relates to or addresses. Facilitators should guide groups to encourage inclusive dialogue and psychological safety. Potential discussion points/answers are displayed below. Remember to keep the conversation centered on the discussion points to provide ample time for the case studies. If the learners overlook any points the facilitator may guide the conversation or state the points.*

| **Discussion Questions** | **Potential Discussion Points** |
| --- | --- |
| How would you have felt if you were in Dr. Brooks’ shoes?  Facilitators should note that this question aligns with the **Gibbs Reflective Cycle – “Feelings” stage**, which focuses on identifying initial emotional responses before moving into evaluation or analysis.^20, 21^ | - Facilitators can invite participants to engage in self-reflection, emphasizing them to think through how each isolated event could have affected Dr. Brooks’ individually and collectively. Emphasize that bias is a common and often unconscious aspect of human cognition and may not present as an overt or emotionally charged response. Bias can surface subtly or more overtly via microaggressions or encounters with colleagues that can be demeaning. - Labeling expressions of identity—such as natural hair, piercings, or direct communication—as “unprofessional” can marginalize trainees and reinforce exclusionary standards. These judgments may lead to self-censorship, reduced engagement during clinical activities, erosion of confidence, and increased emotional burden. Over time, this can contribute to burnout, disengagement, and attrition of trainees from underrepresented backgrounds, ultimately affecting workforce diversity and patient care. |
| Think about the narrative that you are creating about Dr. Brooks. Which of your biases could be rooted within that narrative?  Facilitators should note that this question aligns with the **Gibbs Reflective Cycle – “Analysis” stage**, which encourages participants to make sense of the situation and explore why the events happened as they did.^20, 21^ | - Participants should be encouraged to really reflect on the narrative that they are creating about Dr. Brooks and who she is based on this scenario. They should identify potential biases that they have about her and how the commentary that other colleagues used to describe her plays into their narrative. Do they think there are truths behind these descriptive terms? Consider how these assumptions may shape her clinical assessments, communication, and care delivery. |
| If a colleague shares with you that they have experienced something similar to what Dr. Brooks experienced, how would you support them?  Facilitators should note that this question aligns with the **Gibbs Reflective Cycle – “Conclusion” stages**, which focuses on identifying opportunities for intervention and considering how future practice may change as a result of reflection.^20, 21^ | - Peers could have actively ensured equitable participation during rounds by minimizing interruptions and amplifying Amina’s clinical insights. Mentors and chief residents could have validated her experiences, acknowledged the impact of microaggressions, and advocated for her within the team. Leadership could have addressed biased feedback. - Encourage participants to reflect on ways that they could implement these aforementioned actions in real time or in a future scenario. |

**Slide 36:** Case #2 Group Reflections

*The facilitator will briefly review the questions aloud. They can also emphasize that the word in bold before the start of each question correlates with the part of the Gibbs Reflective Cycle that the question relates to or addresses. The facilitator will allow audience members to form small groups (groups of 2-6 people depending on the audience size) and have time to reflect on the questions as a group. Following the group reflection, audience members from the different groups can discuss aloud their responses to the reflection questions. Potential discussion points/answers are displayed below. Facilitators should guide audience members to encourage inclusive dialogue and psychological safety.*

*Remember to keep the conversation centered on the discussion points to provide ample time for the case studies. If the learners overlook any points the facilitator may guide the conversation or state the points.*

| **Discussion Questions** | **Potential Discussion Points** |
| --- | --- |
| What thoughts and feelings came up for you while reading this case?  Facilitators should note that this question aligns with the **Gibbs Reflective Cycle – “Feelings” stage**, which focuses on identifying initial emotional responses before moving into evaluation or analysis.^20, 21^ | - The facilitator can encourage participants to pause and reflect on their immediate emotional reactions before moving into analysis or problem-solving. Emphasize that emotions are a meaningful part of clinical reflection and often provide insight into underlying values, assumptions, or discomfort. - Encourage participants to notice a range of responses, including discomfort, shock, frustration, concern, sadness, defensiveness, or even emotional distance. It can be noted that there is no expectation to resolve these feelings, only to acknowledge them as part of the reflective process. |
| What shortcomings did you notice in this case?  Facilitators should note that this question aligns with the **Gibbs Reflective Cycle – “Evaluation” stage**, which emphasizes recognizing what went well and what did not, without assigning blame. This stage helps participants identify gaps and shortcomings in inclusivity, care delivery, and systems.^20, 21^ | - The facilitator can guide participants to identify areas where support for the Dr. Brooks fell short, emphasizing that this was not a single isolated occurrence but a series of repeated scenarios. - Encourage participants to name both interpersonal and systemic shortcomings, including biases, missed opportunities for support following Dr. Brooks’ discussion with the chief, systems-level failures, microaggressions, and the use of labeling language. - Following the discussion, you can point out that what may seem like a small, isolated scenarios can compound and lead to negative encounters for healthcare professionals and ultimately negative health outcomes |
| What sense can you make of the situation? Why do you think this happened? Have you seen similar situations occur in your own clinical settings?  Facilitators should note that this question aligns with the **Gibbs Reflective Cycle – “Analysis” stage**, which encourages participants to make sense of the situation and explore why the events happened as they did.^20, 21^ | - Facilitators can encourage participants to step back and consider the broader context of this situation. This includes reflecting on how bias, microaggressions, how Dr. Brooks might feel and how that could affect her ability to provide care to patients, lack of inclusivity, hierarchy, and institutional norms may influence residents’ (or health care professionals in general’s) experiences - which can ultimately negatively affect patient care. - Participants should be encouraged to connect this case to their own experiences and recognize patterns instead of viewing this as an isolated event. - Facilitators can normalize that many participants may have witnessed or been part of similar situations, and that naming these patterns is an important step toward change. |
| What else could have been done? Who had the opportunity to intervene? When similar situations have occurred in your own clinical settings, did you intervene? Why or why not?  Facilitators should note that these questions align with the **Gibbs Reflective Cycle – “Conclusion” stages**, which focuses on identifying opportunities for intervention and considering how future practice may change as a result of reflection.^20, 21^ | - Facilitators can guide participants to reflect on missed opportunities for intervention at multiple points in time, including individual clinicians, team members, and system processes. Emphasize that intervention does not have to be dramatic or confrontational. - Participants should be encouraged to reflect honestly on barriers to speaking up, such as hierarchy, fear of conflict, uncertainty, or potential career repercussions. Facilitators can invite participants to consider small, practical actions that could be taken in future situations, including slowing down assumptions, advocating for more inclusivity and better understanding of colleagues and their perspectives, addressing microaggressions, or challenging labeling and stereotypical language. |

**Slide 37:** Case #2: Key Takeaways

*The facilitator will share the following key takeaways:
- Bias and microaggressions are common and harmful to trainees.
- Active affirmation and allyship prevent trainee isolation and burnout.
- Program leadership and peers play a key role in fostering an inclusive work environment.*

*The facilitator can also tie in key points and takeaways that were mentioned by audience members and tie them into these key takeaways as they address each individual take away. The facilitator could even mirror language that was used or described by audience members to better personalize and connect the takeaways to the already discussed points from the audience.*

**Data Dictionary Definitions:**

**Cultural Competence:** Cultural competence is characterized as a skill that can be taught, trained, and achieved and is often described as a necessary and sufficient condition for working effectively with diverse patients^1^

**Cultural Humility:** Cultural humility involves entering a relationship with another person with the intention of honoring their beliefs, customs, and values. It entails an ongoing process of self-exploration and self-critique combined with a willingness to learn from others.^2^

**Diversity:** Diversity involves understanding that each individual is unique and recognizing our individual differences. These differences can be along the dimensions of race, ethnicity, ancestry, national origin, immigration status, religion, gender, marital or family status, sexual orientation, gender identity or expression, age, veteran status, disability, or other characteristics.^1,3^

**Explicit Bias:** A belief is explicit if consciously endorsed. Explicit forms of bias include preferences, beliefs, and attitudes of which people are generally consciously aware, personally endorse, and can identify and communicate^4^

**Health Disparities:** Health disparities are largely preventable health differences that adversely affect populations who experience greater challenges to optimal health and are closely linked with intergenerational social, economic, and/or environmental factors—primarily observed among racial and/or ethnic minority populations and/or low socioeconomic status (SES) groups.^5,6^

**Health Equity:** The state in which everyone has a fair and just opportunity to attain their highest level of health. Achieving health equity requires valuing everyone equally with focused and ongoing societal efforts to address avoidable inequalities, historical and contemporary injustices, and the elimination of health and healthcare disparities.^5^

**Implicit Bias:** Implicit bias is the attitude or internalized stereotypes that unconsciously affect our perceptions, actions, and decisions. These unconscious biases often affect behavior that leads to unequal treatment of people based on race, ethnicity, gender identity, sexual orientation, age, disability, health status, and other characteristics.^4,7^

In medicine, unconscious bias-based discriminatory practices negatively impact patient care and medical training programs, hinder effective communication, limit workforce diversity, lead to inequitable distribution of research funding, impede career advancement, and result in carriers and disparities in the access to and delivery of healthcare services.

**Inclusion:** Inclusion represents the engagement and participation of diverse individuals in an organization. Initiatives around inclusion recognize the inherent worth and dignity of all individuals, and work to include all into the organization's overall capacity and decision-making.^1^

**Intersectionality:** How multiple identities interact to create unique experiences of privilege and power^9^

**Microaggressions:** everyday slights, insults, invalidations, and offensive behaviors experienced by individuals from marginalized groups. These actions often occur during interactions with generally well-intentioned individuals, who may be unaware that their behavior is demeaning or harmful.^10^

**References**
1) American Academy of Pediatrics. *“Diversity and Inclusion.”* *American Academy of Pediatrics Equity and Inclusion Efforts*, AAP, www.aap.org/en/about-the-aap/american-academy-of-pediatrics-equity-and-inclusion-efforts/diversity-and-inclusion/
2) Stubbe DE. Practicing Cultural Competence and Cultural Humility in the Care of Diverse Patients. Focus (Am Psychiatr Publ). 2020 Jan;18(1):49-51. doi: 10.1176/appi.focus.20190041. Epub 2020 Jan 24. PMID: 32047398; PMCID: PMC7011228.

3) National Institute on Minority Health and Health Disparities. *“What Are Health Disparities?”* NIMHD, 13 Mar. 2025, www.nimhd.nih.gov/about/what-are-health-disparities

4) Vela MB, Erondu AI, Smith NA, Peek ME, Woodruff JN, Chin MH. Eliminating Explicit and Implicit Biases in Health Care: Evidence and Research Needs. Annu Rev Public Health. 2022 Apr 5;43:477-501. doi: 10.1146/annurev-publhealth-052620-103528. Epub 2022 Jan 12. PMID: 35020445; PMCID: PMC9172268.

5) CNN. “DEI, Diversity, Equity and Inclusion, Explained.” *CNN*, 22 Jan. 2025, https://www.cnn.com/2025/01/22/us/dei-diversity-equity-inclusion-explained.
6) Flores G, Lesley B. Children and US Federal Policy on Health and Health Care: Seen but Not Heard. *JAMA Pediatr.* 2014;168(12):1155–1163. doi:10.1001/jamapediatrics.2014.1701
7) Shah HS, Bohlen J. Implicit Bias. [Updated 2023 Mar 4]. In: StatPearls [Internet]. Treasure Island (FL): StatPearls Publishing; 2025 Jan-. Available from: https://www.ncbi.nlm.nih.gov/books/NBK589697/

8) Samie, A. (2025, December 11). intersectionality. Encyclopedia Britannica. https://www.britannica.com/topic/intersectionality

9) Mosley, T. J., Zajdel, R. A., Alderete, E., Clayton, J. A., Heidari, S., Pérez-Stable, E. J., Salt, K., & Bernard, M. A. (2025). Intersectionality and diversity, equity, and inclusion in the healthcare and scientific workforces. Lancet regional health. Americas, 41, 100973. https://doi.org/10.1016/j.lana.2024.100973

10) University of Colorado Boulder, Center for Teaching & Learning. “Microaggressions.” *Center for Teaching & Learning – Inclusivity*, Colorado.edu, www.colorado.edu/center/teaching-learning/inclusivity/microaggressions

11)James M. Perrin, Patricia Flanagan, Julie Katkin, Greg Barabell, Jonathan Price, Committee on Child Health Financing; The Unique Value Proposition of Pediatric Health Care. *Pediatrics* February 2023; 151 (2): e2022060681. 10.1542/peds.2022-060681

12) Katz VS, Gonzalez C, Clark K. Digital Inequality and Developmental Trajectories of Low-income, Immigrant, and Minority Children. Pediatrics. 2017 Nov;140(Suppl 2):S132-S136. doi: 10.1542/peds.2016-1758R. PMID: 29093048.
13) Jenkins, Richard. *Social Identity*. 4th ed., Routledge, 2014.

14) The Commonwealth Fund. (2023, July 19). The case for diversity in the health professions remains powerful. <https://www.commonwealthfund.org/blog/2023/case-diversity-health-professions-remains-powerful>
15) Leonard Davis Institute of Health Economics at the University of Pennsylvania. (2025, April 29). Patients do better when care teams collaborate. <https://ldi.upenn.edu/our-work/research-updates/patients-do-better-when-care-teams-collaborate/>
16). Gomez LE, Bernet P. Diversity improves performance and outcomes. J Natl Med Assoc. 2019 Aug;111(4):383-392. doi: 10.1016/j.jnma.2019.01.006. Epub 2019 Feb 11. PMID: 30765101.

17) Machost, H., & Stains, M. (2023). Reflective Practices in Education: A Primer for Practitioners. CBE life sciences education, 22(2), es2. <https://doi.org/10.1187/cbe.22-07-0148>

18) Kumagai AK, Lypson ML. Beyond cultural competence: Critical consciousness, social justice, and multicultural education. Acad Med. 2009;84(6):782–787.
19) Acosta D, Ackerman-Barger K. Breaking the silence: time to talk about race and racism. Acad Med. 2017;92(3):285–288.

20) The Open University. *“6.2 Gibb’s Reflective Cycle.”* *Learning to Teach: Becoming a Reflective Practitioner*, OpenLearn, The Open University, https://www.open.edu/openlearn/education-development/learning-teach-becoming-reflective-practitioner/content-section-6.2.

21) The University of Edinburgh. “Gibbs’ Reflective Cycle.” Reflection Toolkit — Reflectors’ Toolkit: Reflecting on Experience, The University of Edinburgh, <https://reflection.ed.ac.uk/reflectors-toolkit/reflecting-on-experience/gibbs-reflective-cycle>. Accessed 8 Jan. 2026. ([Reflection Toolkit](https://reflection.ed.ac.uk/reflectors-toolkit/reflecting-on-experience/gibbs-reflective-cycle?utm_source=chatgpt.com))

Note, additional dictionary definition terms are explicitly defined in the Facilitator Guide, and those references were included within this appendix.
