# Supplementary material for: From Reflection to Action: A Reflective Learning Workshop to Bridge Gaps in Pediatric Health
Source: MedEdPORTAL. 2026 Nov 10;22:11651. doi: 10.15766/mep_2374-8265.11651 (PMC13749098; doi:10.15766/mep_2374-8265.11651)
Supplement: Supplementary file 1 [file mep_2374-8265.11651-s001.zip › E. Reflection Document for Participants.docx]

**Appendix E**

**From Reflection to Action: A Reflective Learning Workshop to Bridge Gaps in Pediatric Health**

**The Gibbs Reflective Cycle**

Gibbs Reflective Cycle. Adapted from Gibbs G. Learning by Doing: A Guide to Teaching and Learning Methods. Oxford Polytechnic; 1988.

**Cases**

**Case 1:**

A 5-year-old boy is brought to the ED by his father for lethargy and vomiting. The father has limited English proficiency and smells faintly of marijuana. The resident documents the odor, assumes accidental ingestion, and orders a urine toxicology screen before fully examining the child. The family is placed in an observation room. The interpreter phone is broken. No in-person interpreter is available, and no effort is made to find one.

Hours later, the child becomes hypotensive. Another provider discovers asymmetric pupils and neck stiffness—findings missed on the initial encounter. The family, though distraught, remains calm and asks why no one listened earlier. In sign-out, the resident says, “That family’s always defensive. They’re covering up something,” and warns others to expect a defensive family. A team member even suggests flagging them in the EMR for future safety concerns.

**Case 1: Self Reflection Questions**

1) **Description:** How would you describe what just occurred?
2) **Feelings:** What explicit or implicit biases did you feel coming up for you?
3) **Evaluation and Analysis:** How would you have displayed cultural humility in this scenario?
4) **Action:** Following the completion of this self-reflection, how will you change how you practice in the future?

**Case 1: Group Reflection Questions**

1) **Feelings:** What thoughts and feelings came up for you while reading the case?
2) **Evaluation:** What shortcomings did you notice in this case?
3) **Analysis:** What sense can you make of this situation? Why do you think this happened? Have you seen similar situations occur in your own clinical settings?
4) **Conclusion and Action:** What else could have been done? Who had the opportunity to intervene? When similar situations have occurred in your own clinical settings, did you intervene? Why or why not?

**Case 2:**

Dr. Amina Brooks, a female Afro-Latina pediatric resident, has grown increasingly quiet during team rounds. She used to be excited to work at a hospital that serves a majority Black and Latinx population but now finds herself constantly navigating small microaggressions. She wears her natural hair in styles such as an afro, braids, or twists. She once overheard a fellow whisper, “She’s always doing something different with her hair.” During presentations, she is interrupted more often than her peers and her teaching points are often dismissed as “too detailed”. This rarely happens with other trainees. Amina has started writing notes to reassure herself that she’s not imagining things.

When she shares her discomfort with a chief resident, her concerns are brushed aside that “perception is everything” and that she should “read the room.” She’s later described in a feedback session as “quiet, but intense” and “not very approachable.” Amina begins to question whether she belongs and whether she can thrive in an institution where no one above her looks like her or affirms her presence.

**Case 2: Self Reflection Questions**

1) **Feelings:** How would you have felt if you were in Dr. Brooks’ shoes?
2) **Analysis:** Think about the narrative that you are creating about Dr. Brooks. Which of your biases could be rooted within that narrative?
3) **Conclusion:** If a colleague shares with you that they have experienced something similar to what Dr. Brooks experienced, how would you support them?

**Case 2: Group Reflection Questions**

1) **Feelings:** What thoughts and feelings came up for you while reading the case?
2) **Evaluation:** What shortcomings did you notice in this case?
3) **Analysis:** What sense can you make of this situation? Why do you think this happened? Have you seen similar situations occur in your own clinical settings?
4) **Conclusion:** What else could have been done? Who had the opportunity to intervene? When similar situations have occurred in your own clinical settings, did you intervene? Why or why not?

**Creating an Action Plan**

Kern’s Six Step Model, Adapted Kern, David E., et al. Curriculum Development for Medical Education: A Six-Step Approach. 2nd ed., Johns Hopkins University Press, 2009.
